# Editorial: The Role of Hematopoietic Progenitors in Immune Regulation and Memory

**DOI:** 10.3389/fimmu.2021.789139

**Published:** 2021-11-12

**Authors:** Flora Zavala, César Nombela-Arrieta, Moufida Ben Nasr, Paolo Fiorina

**Affiliations:** ^1^ Université de Paris, INSERM U1151, CNRS UMR8253, Institut Necker Enfants Malades, Paris, France; ^2^ Department of Medical Oncology and Hematology, University of Zurich and University Hospital Zurich, Zurich, Switzerland; ^3^ International Center for Type 1 Diabetes, Pediatric Clinical Research Center Romeo ed Enrica Invernizzi, Department of Biomedical and Clinical Science, Università di Milano, Milan, Italy; ^4^ Nephrology Division, Boston Children’s Hospital, Harvard Medical School, Boston, MA, United States; ^5^ Division of Endocrinology, Territorial Healthcare Company Fatebenefratelli-Sacco, Milan, Italy

**Keywords:** hematopoietic progenitors, transplantation, immune memory, hematopoietic niche, stromal cells, trained immunity, autoimmunity, graft versus host disease (GVHD)

Hematopoietic stem cells and progenitors (HSPCs) represent an indispensable reservoir for the continuous replenishment of all immune and blood cells. HSPCs mostly reside within the bone marrow (BM) microenvironment in close interaction with a variety of stromal cell types that provide a regulatory infrastructure that controls quiescence or multilineage differentiation through the provision of instructive signals. The first part of this Research Topic focuses on the intricate nature of the cellular crosstalk between HSPCs and their niche, specifically, i) the functional and spatial complexity of hematopoietic niches, ii) the effects of infectious and inflammatory signals on the integrity of niches and hematopoiesis. The second set of articles explores the evidence suggesting that stimulation of HSPCs by various inflammatory or infectious signals can promote/enhance their trafficking and interaction with mature immune cells in peripheral tissues (1, 2), where they exert either an immune-enhancing effect or, conversely, an immunoregulatory effect on initiating or ongoing immune responses. Finally, several research papers characterize selective hematopoietic progenitor subsets with immunoregulatory properties *in vitro* as well as in experimental models of infection, autoimmune and alloimmune responses.

The complexity and overlapping roles of the hematopoietic and immune cell niches are reviewed in detail by Miao et al. The authors cast a special focus on the CXCR4/CXCL12 axis as a core pathway controlling quiescence and access of HSPCs to their niches and highlight the key functional roles of CXCL12-producing mesenchymal stromal cells (MSCs), in the replenishment of mature components of innate immunity in homeostasis as well as during stress. As the most prominent source of key cytokines instructing lineage specification, survival, and long-term maintenance of HSPCs, perturbations of their structural and functional integrity (3) underlie prototypical features of hematopoietic responses to infection and inflammation. As prime examples, the authors analyze the disruptive effects that WHIM syndrome, a combined immunodeficiency disease caused by a genetic mutation in the chemokine receptor gene CXCR4, myeloablative irradiation and leukemia, trigger in HSPC niches and the stromal infrastructure through the activation of a proinflammatory program. Such insults are known to cause defects in hematopoietic cell development and recirculation, leading to immune deficiency and favoring malignant transformation.

In two related articles, Sezaki et al. as well as Johnson et al., describe how infectious challenges or, conversely, antibiotic treatments affect hematopoiesis and the BM microenvironment. A number of recent exciting findings are highlighted in this context. Among them i) the emerging role of microbiota in fine-tuning hematopoiesis through the effects of circulating microbial molecules on BM resident hematopoietic and stromal cells ii) evidence suggesting the contribution of emergency granulopoiesis to anti-tumoral immunity (4), iii) the potential role of infection-mediated mobilization of HSCs from the BM through pathways involving downregulation of CXCL12 to the replenishment of empty niches in distal bones, iv) the detrimental effects of infections of stromal cells, such as the observed depletion of osteoblasts during sepsis, which leads to inefficient lymphopoiesis because of insufficient IL-7 production. Of major interest is the discussion on breakthroughs in our understanding on how microbial-dependent inflammation educates HSCs, induces a bias towards the myeloid lineages, and leads to the generation of monocytes and macrophages, presenting a primed state of hyper-responsiveness that enhances their innate immune function upon subsequent challenge. This type of unspecific, innate memory, termed trained immunity, is imprinted at the epigenetic and metabolic level in HSPCs, and has attracted major attention in recent times (5–7). Conversely, these reviews also describe studies showing how immunoregulatory properties are instructed by a variety of innate signals and pharmacological agents on specific HSPC subsets. This phenomenon could be fundamentally exploited for the control of immune responses by resetting an aberrant autoreactive immune system to a *de novo* self-tolerant immune system (8, 9).

Pastore et al. review the defects in HSC characterizing the murine model of spontaneous type 1 diabetes in the Non-Obese Diabetic (NOD) mouse, in which high CXCL12 BM levels alter the trafficking of HSCs and Tregs and favor T1D onset. The mixed-chimerism induced by HSC infusion re-established autoreactive thymic T-cell deletion and delayed T1D onset. Moreover, infusion of ex vivo genetically engineered HSPCs, for instance, to express proinsulin or transgenically target MHC class II, successfully prevented T1D onset in NOD mice by reshaping the immune reservoir and facilitating tolerance towards auto-antigens. They further review clinical trials of HSCT in new-onset T1D patients (9, 10) that conferred insulin independence for 4-6 years after HSCT and particularly delineated a selective subgroup of patients with a different immune profile that may benefit the most from AHSCT. However, HSCT required myeloablation, which may limit its clinical application. The alternative infusion of pharmacologically modulated or genetically engineered HSPCs, avoiding myeloablation, is advocated (9, 11–14).

Finally, several papers describe selective immature progenitor subsets endowed with immunoregulatory properties.

Marinescu et al. describe a BM subset contaminating MSCs at initial culture passages that exhibits a phenotype of anti-inflammatory macrophages and inhibits T-cell proliferation *in vitro*, but, unlike BM MSCs, does not exert anti-tumoral effects *in vivo*.

Elahi et al. and Mashhouri et al. focus on CD71^+^ erythroid progenitors, a prominent source of Reactive Oxygen Species (ROS) production in both mice and humans, more abundant in female than in male, with immunosuppressive properties that compromise immune response against systemic *Listeria monocytogenes* infection in neonatal mice. Their data underline the tight regulation of the immune system in newborns/infants.

Korniotis et al. and D’Aveni et al. focus on G-CSF mobilized multipotent MPP3 progenitors that display the property to selectively promote the proliferation of TCR-activated Foxp3^+^ Tregs *in vitro* and *in vivo*. This underlines their capacity to reduce autoimmune encephalomyelitis (EAE), a murine model of multiple sclerosis. Additionally, sustained beneficial effects comprised prevention of the onset of T1D in NOD mice (15) and also a reduction of Graft-*versus*-Host Disease (GVHD), a deleterious complication of allogeneic HSC transplantation observed in patients with hematological malignancies. The human counterpart of this suppressive mobilized MPP subset is characterized.

Overall, the contributions in this Research Topic focus on the use of *ex vivo* conditioned HSPCs as a potentially safer therapy than AHSCT, minimizing/eliminating the toxic conditioning that infers unacceptable risks for autoimmune patients. HSPCs have successfully rendered patients suffering from autoimmune diseases, disease-free. In addition, they may as well reduce the severity of GVHD post allogeneic HSCT in patients with hematological malignancies.

## Author Contributions

All authors contributed equally to the redaction of the research focus. All authors have seen and agree with the final version.

## Funding

FZ was supported by core funding from CNRS and INSERM and by grants received from Fondation pour la Recherche sur la Sclérose en Plaques (ARSEP) and from The Secular Society (TSS). CN-A is supported by Swiss National Science Foundation (310030_185171). PF was supported by the Italian Ministry of Health grant RF-2016-02362512.

## Conflict of Interest

The authors declare that the research was conducted in the absence of any commercial or financial relationships that could be construed as a potential conflict of interest.

## Publisher’s Note

All claims expressed in this article are solely those of the authors and do not necessarily represent those of their affiliated organizations, or those of the publisher, the editors and the reviewers. Any product that may be evaluated in this article, or claim that may be made by its manufacturer, is not guaranteed or endorsed by the publisher.
